# Peripheral inflammation and blood–brain barrier disruption: effects and mechanisms

**DOI:** 10.1111/cns.13569

**Published:** 2020-12-30

**Authors:** Xiaowen Huang, Basharat Hussain, Junlei Chang

**Affiliations:** ^1^ Shenzhen Key Laboratory of Biomimetic Materials and Cellular Immunomodulation Institute of Biomedicine and Biotechnology Shenzhen Institute of Advanced Technology Chinese Academy of Sciences Shenzhen China; ^2^ University of Chinese Academy of Sciences Beijing China

**Keywords:** blood–brain barrier, central nervous system, inflammation, inflammatory factors, molecular mechanism

## Abstract

The blood–brain barrier (BBB) is an important physiological barrier that separates the central nervous system (CNS) from the peripheral circulation, which contains inflammatory mediators and immune cells. The BBB regulates cellular and molecular exchange between the blood vessels and brain parenchyma. Normal functioning of the BBB is crucial for the homeostasis and proper function of the brain. It has been demonstrated that peripheral inflammation can disrupt the BBB by various pathways, resulting in different CNS diseases. Recently, clinical research also showed CNS complications following SARS‐CoV‐2 infection and chimeric antigen receptor (CAR)‐T cell therapy, which both lead to a cytokine storm in the circulation. Therefore, elucidation of the mechanisms underlying the BBB disruption induced by peripheral inflammation will provide an important basis for protecting the CNS in the context of exacerbated peripheral inflammatory diseases. In the present review, we first summarize the physiological properties of the BBB that makes the CNS an immune‐privileged organ. We then discuss the relevance of peripheral inflammation‐induced BBB disruption to various CNS diseases. Finally, we elaborate various factors and mechanisms of peripheral inflammation that disrupt the BBB.

## 1 INTRODUCTION

The circulatory system contains blood vessels that distribute blood with nutrients and oxygen and remove waste products and CO_2_ from the tissue. Its normal function is essential for maintaining homeostasis of the organism. The inner layer of blood vessels is made of vascular endothelial cells (ECs).^1^ The endothelium is distinct in structure and function, and can be continuous non‐fenestrated, continuous fenestrated, or discontinuous dependent on the organ requirements.^2^ The brain and spinal cord comprise central nervous system (CNS) that controls critical functions of the body. CNS vasculature has a unique anatomy and physiology making the CNS a so‐called “immune‐privileged” organ, although this idea was challenged in the past several decades.^3^ Located between the CNS tissue and peripheral blood circulation, the blood–brain barrier (BBB) regulates cellular and molecular exchange between the blood vessels and brain parenchyma. ECs, pericytes, and astrocytes are the major components of the BBB, and basement membrane between them is also required for the BBB function and integrity.^1^

An important function of the BBB is to maintain the homeostasis of the central nervous system (CNS). BBB dysfunction is implicated in various neurological diseases, such as Alzheimer's disease (AD), Parkinson's disease (PD), amyotrophic lateral sclerosis (ALS), multiple sclerosis (MS), and stroke.^4^ It was recently reported that in patients with autoimmune diseases such as rheumatoid arthritis, treatment with tumor necrosis factor (TNF) inhibitor increased the risk of CNS inflammation and subsequent BBB breakdown.^5^ In addition, patient infected with SARS‐CoV‐2 or those using chimeric antigen receptor (CAR)‐T cell therapy for lymphocytoma could also develop CNS complications, probably due to BBB disruption induced by peripheral inflammation, although definitive conclusion is not drawn yet. Peripheral inflammation refers to the activation of the innate or adaptive immune system and release of proinflammatory cytokines against various pathological stimuli outside of the CNS. It is normally a kind of protective response of the body against multiple insults. Since the BBB is highly susceptible to the inflammatory stimuli, inappropriate peripheral inflammation such as lipopolysaccharide (LPS) can impact the BBB function via different pathways.^6^, ^7^, ^8^

In this review, we briefly describe current understandings on BBB structure and functions. Particularly, we elaborate the most recent advances in mechanisms of BBB disruption secondary to peripheral inflammatory conditions, which have been largely overlooked in the research of CNS diseases.

## 2 STRUCTURE AND CONSTITUENTS OF THE BBB

**FIGURE 1:**
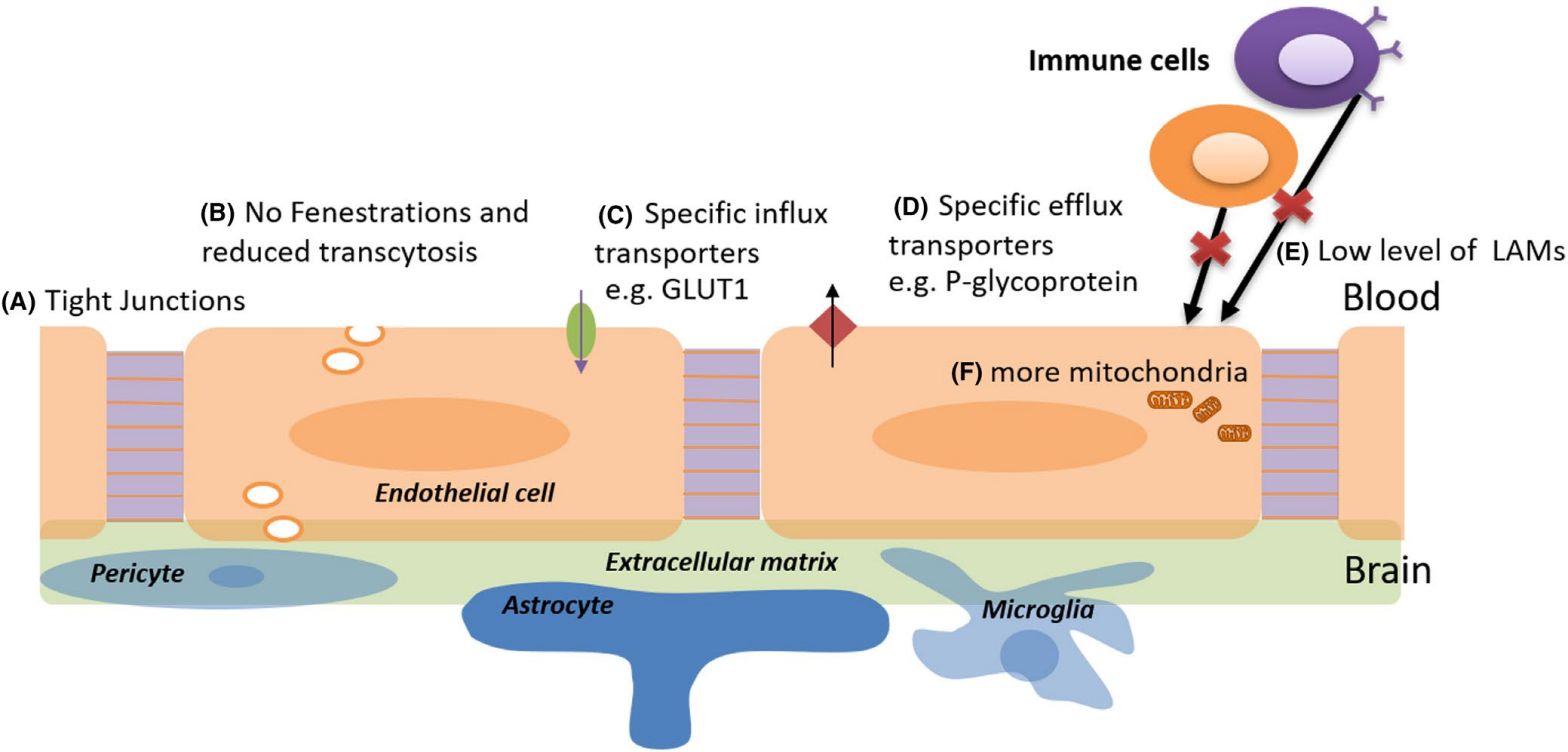
Schematic diagram of the physiological characteristics of the BBB. GLUT1, glucose transporter 1; LAMs, leukocyte adhesion molecules

The neurovascular unit (NVU) usually consists of endothelial cells, mural cells (i.e., vascular smooth muscle cells and pericytes), basement membrane, glia cells (astrocytes and microglia cells), and neurons, which collectively contribute to BBB integrity.^9^ ECs form the inner lining of all blood vessels. BBB ECs are quite different in structure and function from those in other tissues, and the typical characteristics that distinguish them from other ECs include the following: (a) paracellular transport of solutes is blocked due to tight junctions, (b) fenestrations are absent and transcytosis are reduced, limiting transcellular transport of solutes, (c) for transfer of required solutes from the blood to parenchymal cells of the brain, specific transporters, such as GLUT1 (glucose transporter 1), are expressed, (d) to remove toxic substances from CNS parenchymal cells, specific pumps, such as P‐glycoprotein (P‐gp), are expressed, (e) the low expression level of leukocyte adhesion molecules (LAMs) in BBB endothelial cells helps restrict entry of immune cells into the CNS, and (f) ECs of the BBB harbor more mitochondria than of other tissues, which might be related to providing the energy that ionic transport requires (Figure 1).^4^, ^10^, ^11^, ^12^, ^13^ These features of the CNS ECs contribute to highly selected movement of solutes in and out of the CNS parenchyma, maintaining a stable microenvironment for proper neuronal function.^14^

Pericytes cover the CNS capillaries and regulate vascular stability, diameter, cerebral blood flow, and extracellular membrane protein secretion.^15^ Astrocytes span around the vascular endothelium and pericytes via end‐feet, and they are in contact with neurons and regulate BBB permeability.^16^, ^17^ Microglial cells are a component of the NVU, but the link between microglial cells and BBB ECs and its effect on formation and regulation of BBB properties remains to be fully explored. ^18^ Furthermore, the BBB is composed of non‐cellular elements, the extracellular membrane (ECM). All of these cellular and non‐cellular components together maintain the BBB structural and functional integrity (Figure 1).

## 3 BBB FUNCTION AND ITS REGULATION

The complex cellular and non‐cellular components of the BBB collaboratively maintain the BBB function, and impairment in any one of these elements can lead to BBB disruption.^3^ For example, the endothelial tight junctions (TJs) and lack of fenestrae contribute to a physical barrier, leaving carriers and/or receptors the only means for protein transportation into the CNS.^19^, ^20^ ECs play an important role in vascular biology, such as maintaining permeability, homeostasis, and vessel wall integrity and preventing thrombosis.^3^ Pericytes help preserve the TJs of ECs (e.g., Claudin‐5, Occludin, and ZO‐1) and regulate transcytosis in ECs, maintaining the integrity and normal permeability of the BBB.^19^, ^21^, ^22^ Astrocytes secrete factors that are key to maintain BBB properties, including sonic hedgehog (Shh), vascular endothelial growth factor (VEGF), angiopoietins‐1 (Ang‐1), angiotensin‐converting enzyme‐1 (ACE‐1), glial‐derived neurotrophic factor (GDNF), and apolipoprotein E (ApoE).^7^ ECM is a dynamic component of the BBB, and regulates BBB structure and function by impacting cell–cell and cell–matrix interaction within the NVU.^23^ Microglia cells are known as the immune cells in the CNS. They can be activated and categorized into two opposite types: M1 and M2, which produce either cytotoxic or neuroprotective effects.^18^ Under inflammatory conditions, they can be activated to M1 or M2 phenotype, thus damaging or protecting the BBB integrity.^24^, ^25^, ^26^ These cellular and non‐cellular components of NVU are affected directly or indirectly by peripheral inflammation, resulting in disruption of the BBB and CNS alterations.

## 4 PERIPHERAL INFLAMMATION AND CNS DISEASES

### 4.1 Role of BBB disruption in the effects of peripheral inflammation on CNS diseases

Both preclinical and clinical studies have found that peripheral inflammation in the form of infection is a common contributing factor for the development and deterioration of CNS diseases, such as neurodegenerative diseases AD, PD, MS, and stroke. A possible explanation is that BBB disruption in infections increases the susceptibility to CNS diseases.^27^ In AD patients, peripheral inflammation increases the level of amyloid beta (β‐amyloid) in the brain.^28^ In amyloid precursor protein (APP) transgenic mice, peripheral injection of LPS increased BBB permeability, allowing for infiltration of peripheral proinflammatory factors such as IL‐6 and TNF‐α, and promoting neurological inflammation and disease progression.^29^, ^30^ LPS‐induced BBB disruption also plays an important role in the transmission of Tau, probably in a non‐microglia‐dependent pathway.^31^ Aside from AD, evidence also showed peripheral inflammation as a potential risk factor for PD and other neurodegenerative disease.^32^, ^33^ Likewise, dysregulated systemic inflammation is present in PD, as evidenced by high levels of IL‐1β, IL‐2, TNF‐α, CD4^+^ and CD8^+^ T lymphocytes in both serum and cerebrospinal fluid.^34^ In the pathogenesis of MS, one of the most important mechanisms is the infiltration of autoreactive CD4^+^ T cells and other white cells into the CNS, whereas the degree of BBB destruction in experimental autoimmune encephalomyelitis (EAE) model is strongly correlated with disease severity.^35^

Ischemic and hemorrhagic stroke also presents with BBB disruption, and experimental models and clinical observations together have shown that peripheral inflammation (e.g., LPS, anaphylaxis, and infection) is more likely to aggravate BBB disruption and even worsen the outcome of stroke.^36^, ^37^, ^38^, ^39^ For example, the adaptive immune system is activated following cerebral ischemia, and the peripheral immune cells, such as T cells and B cells, rapidly infiltrate the diseased brain and release various cytokines, including pro‐inflammatory cytokines (TNF‐α, IL‐1β, IL‐6) leading to blood vessels and BBB damage, and anti‐inflammatory cytokines (IL‐13, IL‐10, IL‐4, TGF‐β) extenuating the ischemic injury.^40^, ^41^, ^42^ Moreover, both immune cells and cytokines induce immunodepression after stroke, which leads to an increased incidence of infections such as pneumonia.^43^, ^44^, ^45^ It is not exactly clear whether BBB dysfunction induced by inflammation is the cause or complication of CNS disease, and further study to understand the role of peripheral inflammation on BBB function and the influence on CNS disease can provide a basis for clinical treatment of the disease to a certain extent.

### 4.2 BBB disruption in COVID‐19‐related CNS symptoms

In December of 2019, a case of pneumonia caused by a novel coronavirus, SARS‐CoV‐2, emerged in Wuhan, China, and rapidly spread around the world. This new disease is termed coronavirus disease 2019 (COVID‐19) by the World Health Organization (WHO). The most common symptoms of COVID‐19 are fever, cough, and tiredness.^46^ As for its CNS symptoms, according to a retrospective, observational case series of 214 patients, 24.8% of them had CNS manifestations, including ataxia, impaired consciousness, dizziness, and headache.^47^ The most severe cases were 4 with ischemic stroke and 1 with cerebral hemorrhage who died of respiratory failure.^47^ Inflammatory storm is considered one of the causes of death in severe and critical COVID‐19 cases, with over half of which have lymphopenia and a cytokine storm.^48^ Consistently, an increased release of cytokines (IL‐1β, IL1RA, IL‐6, TNF‐α) and chemokines (CCL2, CCL3, CCL5) occurred after infection.^49^, ^50^, ^51^ Conceivably, anakinra (IL‐1 blockade) and tocilizumab (IL‐6 receptor blockade) are showing significant survival benefits in COVID‐19 patients with hyperinflammation.^52^

Although the incidence of CNS complications is high in SARS‐CoV‐2 infections, the pathogenesis is barely known for now. Some researchers believe the cytokine storm during infection persistently affects the CNS.^53^ It is highly likely that BBB disruption might play an important role in the CNS complications associated with COVID‐19.^54^ However, more solid and direct evidence is needed to prove it.

### 4.3 BBB disruption in CAR‐T therapy‐associated neurotoxicity

Chimeric antigen receptor (CAR)‐T cell therapy is a rapidly developing novel strategy for acute lymphoblastic leukemia (ALL) or chronic lymphocytic leukemia (CLL).^55^, ^56^, ^57^, ^58^ Currently approved CAR‐T therapies targeting CD19 showed profound therapeutic effects in ALL.^59^

However, the toxic effects of CAR‐T cells are worrying. The most important and common toxic effects are cytokine release syndrome (CRS) and the associated neurotoxicity, with the most severe of which being lethal cerebral edema.^59^, ^60^, ^61^, ^62^, ^63^, ^64^ CD3^+^ T cells, CD19^+^ B cells, and high levels of cytokines (IFN‐γ, IL‐6) were detectable in the cerebrospinal fluid (CSF) in ALL patients complicated with cerebral edema as soon as a few hours after CD19 CAR‐T cell infusion. This was accompanied by cerebral CRS, probably due to the cytokines produced by BBB‐penetrating CAR‐T cells.^65^ Moreover, cytokines such as IL‐6, IFN‐γ, and TNF‐α were known to directly activate endothelial cells. Patients with severe neurotoxicity showed evidence of endothelial activation, characterized by increased BBB permeability, serving as another important mechanism for neurotoxicity in CAR‐T cell treatment.^66^, ^67^ More recently, researchers reported high CD19 expression in human brain mural cells, but not in mouse mural cells, and that is a possible on‐target mechanism for CD19 CAR‐T cell‐mediated neurotoxicity, meanwhile suggesting limitations in preclinical animal models of neurotoxicity.^68^

Thus far, the mechanisms through which CAR‐T cells cause BBB dysfunction and neurotoxicity remain enigmatic. Nevertheless, it is believed to be closely related to the peripheral inflammatory responses. More in‐depth studies are needed to increase the safety of CAR‐T cell therapy in clinical applications.

## 5 MECHANISMS OF PERIPHERAL INFLAMMATION‐INDUCED BBB DISRUPTION

**FIGURE 2:**
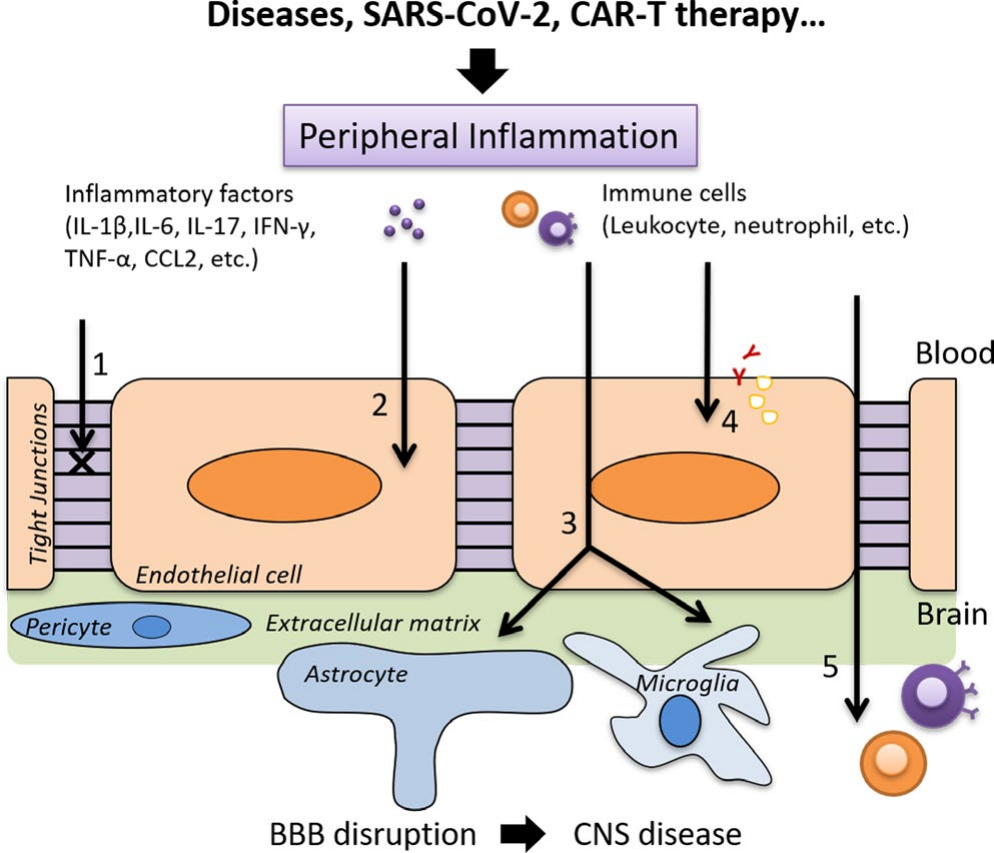
Mechanisms of BBB disruption induced by peripheral inflammation. 1 Changes in tight junctions; 2 damage to endothelial cells; 3 activation of astrocytes and microglia; 4 alteration of multiple transport pathways and receptors; 5 penetration of peripheral immune cells

Peripheral inflammation is basically a protective response for the organism. However, excessive and dysregulated inflammation leads to adverse effects. For example, various non‐neurological systemic infections often come with CNS dysfunction, such as pneumonia and urinary systemic infection, which may be a result of chronic CNS disease.^69^, ^70^ The BBB protects the CNS from potential peripheral insults; therefore, damaging the BBB is considerably harmful to the CNS. Discussed below are mechanisms on how peripheral inflammation impacts the BBB (Figure 2).

### 5.1 Changes in tight junctions (TJs)

TJs are vital components that maintain BBB integrity and normal functioning, such that TJ changes directly lead to BBB disruption. Lots of bacterial and viral infections cause degradation or disorganization of TJs indirectly through diverse pathways.^71^, ^72^, ^73^ For example, cytokines including IL‐1β, IL‐6, IL‐9, IL‐17, IFN‐γ, TNF‐α, and CCL2, can lead to reduced TJ expression or false TJ allocation.^74^, ^75^, ^76^, ^77^, ^78^, ^79^ Claudin‐5 among others is the most important TJ protein responsible for selective permeability of the BBB, and inflammation leads to its downregulation and BBB disruption.^80^ In old mice, LPS‐mediated peripheral inflammation resulted in the degeneration of TJ proteins, including claudin‐5.^81^ On the other hand, IL‐1β led to a discontinuous distribution of claudin‐5 along the plasma membrane of brain endothelial cells.^75^ Apart from claudin‐5, LPS‐induced systemic inflammation was also associated with degradation of occludin.^82^ Another recent study showed that peripheral cytokines reduced expression of ZO‐1 in mice with pre‐existing tumors.^83^

Nowadays, changes in TJs are usually used as indicators of BBB dysfunction. However, there are indirect causes for changes in TJs, such as MMPs, nitric oxide (NO), reactive oxygen species (ROS), Rho‐kinase (ROCK), and NF‐κB signaling pathways.^84^, ^85^, ^86^, ^87^, ^88^ The specific mechanisms will be discussed below.

### 5.2 Damage to endothelial cells

As the primary component of BBB, EC is another important target of peripheral inflammation. Research has shown that LPS has a direct toxic effect on the BBB endothelium by inhibiting P‐gp activity and inducing secretion of MMPs, resulting in membrane abnormalities, endoplasmic reticulum (ER) stress, and mitochondrial damage, and eventually, cell apoptosis.^89^, ^90^ MAPK signaling also contributes to LPS‐induced EC apoptosis.^91^ EC is breakdown, and BBB impairment further facilitates the introduction of neurotoxic substances into the CNS, increasing the risk of other diseases.^89^, ^92^ Another consequence of peripheral inflammation is the upregulated expression of adhesive molecules on ECs, such as Vascular Cell Adhesion Molecule 1 (VCAM‐1), Intercellular Adhesion Molecule 1 (ICAM‐1), and E‐selectin. This allows for trafficking of peripheral immune cells into the CNS, seen in aging and chronic inflammation.^71^, ^93^, ^94^, ^95^ In addition, IL‐1β is found to induced upregulation of α_5_ integrin‐dependent adhesion of EC, which then disrupts the integrity of BBB through altering cell–cell junctions and cell–matrix adhesion.^75^

### 5.3 Activation of astrocytes and microglia

Astrocytes play a vital role in maintaining BBB integrity and regulating its function. Depending on the immune trigger or the phase of inflammation, they produce either pro‐ or antiinflammatory mediators that affect BBB permeability and infiltration of peripheral immune cells.^96^ Under an inflammatory condition, astrocytes secrete VEGF‐A, which activates the eNOS signaling in ECs and downregulates the expression of occludin and claudin‐5, resulting in easy entry of peripheral lymphocytes into the CNS.^97^, ^98^ It has been reported that during inflammation astrocytes altered claudin‐5 expression likely by upregulating the immune‐related GTPase family M‐1 protein (IRGM‐1) in the EAE mouse model.^99^ In middle cerebral artery occlusion (MCAO) model, researchers found high IL‐9 expression in peripheral blood and IL‐9 receptors on astrocytes, and further study revealed that IL‐9 enhances the permeability of the BBB by promoting the secretion of VEGF‐A from astrocytes.^76^ Peripheral inflammation induced by LPS can cause proliferation and activation of astrocytes, changes in the end‐feet structure and altered expression of other related gene, which collectively and indirectly lead to destruction of the BBB.^100^, ^101^

Microglia cells are part of the NVU, but their interaction with BBB ECs and effects on BBB properties are not well known as mentioned above. Even so, there is evidence that shows inflammation‐activated microglia contribute to BBB disruption.^102^ There are two pathways for microglia activation: the M1 proinflammatory pathway and M2 antiinflammatory pathway.^103^ M1 microglia contribute to BBB dysfunction and vascular “leak” mainly through production of proinflammatory mediators, promotion of immune cell trafficking, and oxidative stress.^18^, ^104^ The proinflammatory signaling in M1 microglia involves toll‐like receptor (TLR)‐4,^25^, ^105^ the IFN‐γ receptor complex,^106^ the granulocyte‐macrophage colony‐stimulating factor (GM‐CSF) receptor,^107^ and COX2.^108^, ^109^, ^110^ The secretion of TNF‐α, IL‐1β, IL‐6, IL‐12, CCL2, and CXCL10 is shown to change TJs (claudin‐5, occludin, ZO‐1, and ZO‐2) and critical BBB transporters like P‐gp proteins.^18^, ^26^, ^102^, ^111^, ^112^, ^113^, ^114^ Besides, the chemokines CCL2 and CXCL10 promote trafficking of immune cells cross the BBB, including monocytes and macrophages, which is observed in stroke.^115^, ^116^ In addition to cytokines and chemokines, there is ROS production and oxidative stress in M1 microglia, and it is related to increased expression of iNOS during peripheral inflammation induced by LPS, and also stroke.^117^, ^118^ Different from M1 microglia, M2 microglia play protective roles in BBB disruption, including immune regulation, inflammation dampening, and repair/injury resolution.^119^, ^120^ The polarization to M2 microglia is mediated by IL‐4 receptor, FCγ receptor, IL‐10 receptor, and VEGFR2 signaling.^119^, ^121^ M2 microglia mainly produce antiinflammatory mediators such as TGF‐β1, IL‐10, and IL‐4^122^, ^123^ Receptors for TGF‐β1 are expressed at BBB and TGF‐β1 may have significant positive effects on BBB integrity via activin receptor‐like kinase (ALK)‐1 and −5 signaling.^124^ IL‐10 secretion and IL‐10 receptor promote inflammation suppression and migration of regulatory T cells that alleviate brain injury.^119^, ^125^ In fact, microglia are highly dynamic and their transition from M1 to M2 is complicated and not known clearly. A latest research gave more profound evidence that microglia play a dual role in maintaining BBB integrity in distinct time course of peripheral inflammation.^24^ Initially, brain microglia migrate to cerebral vessels in response to CCL5 and express claudin‐5, thus maintain BBB integrity, whereafter they transform into another phenotype that contributes to BBB leakage.^24^

### 5.4 Effects of peripheral immune cells

Under normal physiological conditions, the BBB restricts the entry of peripheral immune cells into the CNS through low expression of LAMs. However, this is interrupted in pathological conditions. Indeed, peripheral immune cells can have dual roles in BBB integrity depending on different microenvironment and their subtypes, but more evidence show that the infiltration of peripheral immune cells contributes to the disruption of BBB in several neurodegenerative disorders, and even that BBB damage may occur before effector immune cells infiltrate at local sites due to peripheral inflammation‐related or irrelated reasons.^126^, ^127^, ^128^, ^129^ For example, the inflammatory factors secreted by immune cells, such ROS, and MMPs (MMP‐1 and MMP‐2), promote their own migration into the CNS and increase BBB permeability simultaneously, forming a vicious cycle.^74^, ^130^, ^131^

Lymphocytes including T cells (CD4^+^ T helper cells, γδT cells, CD8^+^ cytotoxic T cells), B cells, and NK cells are detrimental to BBB integrity. For T lymphocytes, the interaction of myelin‐reactive CD4^+^ T cells and cerebrovascular ECs plays an important role in regulating BBB integrity. Decisive events in MS and EAE include activation of myelin‐reactive CD4^+^ T cells which then differentiate into effector (Th1 and Th17) and regulatory (Treg) at peripheral tissues, and subsequently transmigrate across the BBB.^132^, ^133^ Th1 and Th17 cells play proinflammatory roles through distinct pathways, while Th2 cells perform antiinflammatory function in stroke (reviewed in Ref. [38]). Th1 cells mainly release proinflammatory cytokines (IL‐2, IFN‐γ, and TNF‐α), promote the transformation of microglia to M1, and mediate cellular immune response.^134^, ^135^ Th17 cells secret IL‐17, IL‐21, and IL‐22 and promote the recruitment of CD4^+^ T cells.^136^, ^137^ IL‐17A (a member of IL‐17 cytokines) activation contributes to BBB disruption by inducing oxidative stress, which then activates the endothelium and downregulates TJ protein occludin.^137^ In addition, peripheral CD8^+^ T cells activation and brain infiltration are detrimental to neural tissue after stroke, in which IL‐2 plays a role.^138^ CD4^+^ and CD8^+^ T cells are found in the brain up to one month post ischemic stroke, and their prolonged activation may affect the outcome of stroke.^139^ But Tregs, as an important subtype of Th2 cells, may be neuroprotective and protect BBB integrity by maintaining immune tolerance, suppressing the activation of other immune cells, or regulating cerebral endothelial function.^39^ Peripheral B cells are a key player in MS on both sides of BBB. They upregulate the activated leukocyte cell adhesion molecule (ALCAM) expression in MS patients and promote the CNS recruitment of monocytes and CD4^+^ T cells, as ALCAM plays a role in BBB integrity for its cell surface localization and association with junctional proteins.^140^, ^141^, ^142^ NK cells are a type of innate immune cells and cytotoxic lymphocytes. It is reported that in cerebral ischemia NK cells produce cytokines such as IFN‐γ, IP‐10, and cause BBB disruption.^71^, ^143^

Other type of immune cells—myeloid cells such as neutrophils, monocytes, dendritic cells (DCs), and mast cells, also influence BBB function via distinct mechanisms. Neutrophils produce a variety of proinflammatory cytokines that affect BBB function, including IL‐1β, TNF‐α, IL‐6, IL‐12, and IFN‐γ, whereas TNF‐α can further induce the recruitment of neutrophils to the CNS. ^144^, ^145^, ^146^ Additionally, when neutrophils transmigrate into the CNS, they secret IL‐1 and activate the antigen‐presenting cells (APC) locally, which subsequently activate endothelial IL‐1R1 signaling that induces T cell recruitment and exacerbates CNS inflammation.^147^ Monocytes may migrate across the BBB depending on the upregulation of cytokines (IL‐1) and junction molecules (ALCAM, JAM‐A, PECAM‐1, and CD99).^148^, ^149^ As a result, HIV‐infected monocytes with upregulation of ALCAM, JAM‐A, and CCR2 on their surface are more likely to cross the endothelium monolayer than noninfected monocytes in response to CCL2, a chemokine, which is elevated in the CNS and CSF of HIV‐infected people.^150^, ^151^ The bone marrow‐derived monocytes (BMDMs) can affect the BBB integrity and control immune infiltration by releasing related cytokines during stroke, whereby exacerbating BBB injury.^127^ Macrophages and DCs are found in the perivascular space between the endothelial and parenchymal basement membranes under inflammation, then help to activate lymphocytes that subsequently breach the BBB.^152^, ^153^, ^154^ The activated mast cells can also produce various proinflammatory mediators, such as histamine, chymase, tryptase, TNF‐α, IL‐6, and IL‐13, which activate MMP‐2 and MMP‐9, thus altering BBB permeability.^155^, ^156^ Actually, myeloid cells, including monocytes, neutrophils, macrophages, and activated microglia, are highly plastic depending on the environment such as interaction with ischemic neurovascular unit during late repair phase of stroke, which can be potential immunotherapeutic targets.^154^, ^157^, ^158^

### 5.5 Other changes in the BBB

In addition to the above pathways in which peripheral inflammation affects the BBB, it is demonstrated that morphological changes may not necessarily occur when peripheral inflammation impacts BBB integrity. For instance, TJs may remain intact during inflammation while the functional integrity of the BBB is impaired.^27^

Multiple transport pathways are altered by peripheral inflammation. Efflux transporters are downregulated, including P‐gp on the astrocytic end‐feet, along with those for anions, amino acids, and β‐amyloid. Meanwhile, influx transporters are upregulated, including those for insulin, monoamine, and lysosomal enzymes.^27^ In addition, the cerebral endothelium expresses IL‐1, IL‐6, and TNF‐α receptors; thus, these circulating cytokines can directly activate the endothelium, causing BBB dysfunction.^159^ This may be associated with nuclear transcription factor IκB.^160^ What is more, LPS, TNF‐α, and IL‐1β can enhance the expression of cyclooxygenase (COX) in the cerebral endothelium.^159^ It has been reported that a high dose of LPS causes BBB damage through COX‐dependent pathways.^161^ Recently, it was identified that dynamic changes of CD antigens, such as CD54 and CD106 in brain vessels, allowed for leukocyte migration with and without alterations of other major functional molecules after LPS injection.^162^

## 6 CONCLUSION AND FUTURE PERSPECTIVE

The BBB is a complex CNS structure that precisely regulates the transport of ions, molecules, and cells between the CNS and periphery. It protects the brain from damage and maintains the normal biochemical microenvironment. Peripheral inflammation is one of the comorbid conditions that is involved in BBB breakage and its dysfunction, and its mechanisms are extremely complicated (Table 1). Take SARS‐CoV‐2, for example, the recent COVID‐19 patients showed ischemic stroke and cerebral hemorrhage, highlighting the potentially critical role of BBB disruption by peripheral inflammation. Our lacking in knowledge in understanding it makes it even harder for treatment and prevention of serious CNS complications in COVID‐19 patients. More work is needed to understand the heterogeneity and signaling mechanisms intrinsic to BBB development, maintenance, disruption, and repair. Although some of the molecular and cellular pathways have been reported, it is crucial to identify how these different signaling pathways collaborate with one another during the development and maintenance of the BBB. Answers to these questions could help tell the exact mechanisms of BBB disruption that lead to various neurological diseases.

**TABLE 1:** Mechanisms of peripheral inflammation causing BBB disruption, and the corresponding examples and references.

Mechanisms of peripheral inflammation‐induced BBB disruption	Examples	References
Changes in TJs	Expression and/or location changes in claudin−5, occludin, ZO−1, etc.	71,72,73,74,75,76,77,78,79,80,81,82,84,83,85,86,87,88,92,97,98,99,102
Damage to ECs	ECs apoptosis, membrane abnormalities, ER stress, and mitochondrial damage.	89,90,91
	Upregulation of VCAM−1, ICAM−1, and E‐selectin expression in ECs.	93,94,71,95
	Upregulation of α_5_ integrin‐dependent adhesion.	75
Activation of astrocytes and microglia	Astrocytes: increased secretion of VEGF‐A.	97,98,99,76
	Astrocytes: proliferation, activation, and changes in the end‐feet structure.	100,101
	Microglia: M1 pro‐inflammatory microglia; M2 antiinflammatory microglia.	24,26,102,105,25,106,107,108,109,110,111,112,113,114,115,116,117,118,119, 163 120,121,122,123,124,125
Effects of peripheral immune cells	Migration of peripheral immune cells to CNS promoted by inflammatory mediators (ROS, MMP, etc.).	74,126,130,131
	Effects of lymphocytes on BBB: myelin‐specific CD4^+^ T cells, Th1, Th17 cells, CD8^+^ T cells, Th2 cells (Tregs), B cells, NK cells, etc.	132, 133 38,134,135,136,137 138,139 39 140,141,142 143,71
	Effects of myeloid cells on BBB: neutrophils, monocytes, macrophages, DCs, mast cells, etc.	144,145,146,147 146,148,149,150,127,151 152,153 155,156
Others	Changes in transport pathways: efflux and influx transporters.	27,159,160,161,162
	Peripheral inflammation in CNS diseases (AD, PD, MS, stroke, etc.)	28,29,30,31,32,33,34,35,36,37,40,41,43,44,45,132,133,138
	SARS‐CoV−2 virus infection‐induced peripheral inflammation affecting BBB.	46,47,48,49,50,51,52,53, 54
	Neurotoxic effects of CAR‐T therapy.	59,60,61,62,63,64,65,67,66,68 164

Although it is almost certain that peripheral inflammation can induce BBB dysfunction, it is not clear whether this serves as an etiology for the development of various CNS diseases. Future research to study on this attribute could provide a basis for clinical treatment of the disease. It will also hint the identifications of new therapeutic strategies in various CNS diseases targeting BBB repair. On top of that, it may also shed lights to develop effective strategies for the CNS delivery of drugs to treat a wide range of neurological diseases.

## CONFLICTS OF INTEREST

The author declares no conflict of interest.

## Data Availability

Data sharing is not applicable to this article as no new data were created or analyzed in this study.
